# Wood shadows: The influence of *Xylophaga* on hard-substrate macrofauna in Southern California

**DOI:** 10.1371/journal.pone.0337217

**Published:** 2025-12-03

**Authors:** Ailish Ullmann, Guillermo Mendoza, Michelle Guraieb, Ana Patricia Galindo, Ximena Flores Flores, Lisa A. Levin

**Affiliations:** 1 Integrative Oceanography Division and Center for Marine Biodiversity and Conservation, Scripps Institution of Oceanography, University of California, San Diego, La Jolla, California, United States of America; 2 School of Biological Sciences, Victoria University of Wellington, Wellington, New Zealand; 3 Earth Sciences New Zealand, Wellington, New Zealand; L3 Scientific Solutions, GERMANY

## Abstract

The abundant small taxa on hard substrates in deep-sea environments remain understudied and therefore poorly understood. Abundant seepage, tectonic activity, upwelling, and high productivity generate a diverse array of hard substrates including basalts, carbonate rocks, phosphorites, ferromanganese crusts, and sedimentary rocks throughout the Southern California Borderland (SCB), and wood falls are common given the region’s proximity to land. A comparative experimental approach was used to examine the influence of hard substrate type on patterns of macrofaunal colonization under different environmental conditions, and particularly revealed the outsized influence of the wood-boring bivalve *Xylophaga* on macrofaunal colonization both on and near wood substrates. The experimental substrates––wood (to assess the influence of organic falls), carbonate rock (to assess faunal affinities for seep-related substrates outside of a seep environment), and other rocks (ferromanganese, phosphorites, and sedimentary rocks, which are common throughout the SCB)––were deployed for ten months at two sites (San Juan Seamount, 40-Mile Bank), each at two water depths (~700 m, ~ 1100 m) in the SCB. The settlement of juvenile *Xylophaga* on wood and nearby substrates drove overall density trends and contributed to the distinct community composition observed among substrate types. Experimental substrates exhibited significantly higher densities than natural rocks, possibly representing the influence of different substrate or earlier successional stages. Diversity was highest on natural rock substrates and lowest on experimental wood substrates, which also exhibited the lowest evenness due to dominance by wood specialists such as *Xylophaga*, dorvilleid polychaetes, and the ampharetid polychaete *Decemunciger* sp. Juvenile *Xylophaga* were found on both experimental wood substrates and nearby rock substrates, and likely provided other species with food, and on wood, with modified (engineered) habitat. These findings suggest that natural wood falls could have a shadow effect on macrofauna of adjacent hard substrates in this region, which merits future study.

## Introduction

The Southern California Borderland (SCB) is a region containing basins, ridges, seamounts, knolls, and escarpments that are subject to active tectonics, leading to extensive hardground and heterogeneous geological, biological, and chemical characteristics. Its seafloor is commonly host to wood falls (via inputs from California’s forested coastline [1]), authigenic carbonate rocks (resulting from anaerobic oxidation of methane at seafloor methane seeps [2–4]), and a variety of both mineral-rich (e.g., phosphorites and ferromanganese crusts [5]) and other rocks (e.g., basalts and sedimentary rocks) [6–9]. In light of growing interest from extractive industry in mineral-rich rocks––and with growing human disturbance from pollution to climate change––it is increasingly important to understand the mechanisms by which diverse hard-substrate ecosystems function in order to inform their management and conservation.

Hard substrate colonization experiments conducted at the seafloor offer a means to elucidate these mechanisms and dynamics, and are often used to study succession in wood falls [10–13] and at methane seeps [14–17]. However, habitats effectively isolated from reducing settings have not been as thoroughly studied. Organic fall colonization patterns indicate that these systems may act as stepping stones that connect seep and vent fauna at different sites [12,18–20], whether through larval dispersal and recruitment or through colonization by adult individuals [21,22]. On the deep northeast Pacific margin, colonization studies with wood substrate deployments [23], including one that also deployed carbonate rock substrates [24], revealed the strong influence of environmental controls such as depth, proximity to shore, and oxygen concentrations [23,24]. Though these studies shed light on colonization rates and successional stage dynamics on the northeast Pacific margin, there has not yet been a colonization study outside of methane seep environments that compares wood and carbonate rock with natural rock communities. A recent study of natural SCB hard substrate communities demonstrated a significant influence of both substrate type and depth on macrofaunal community structure [9]. However, there remains a gap in knowledge about how substrate type affects colonization of substrates (e.g., after a disturbance) and how nearby organic falls may influence this colonization through instances of “mass effect,” or spillover of organic specialists from nearby wood falls onto non-wood substrates. This phenomenon has been observed in wood fall and colonization studies before [13,23]. However, since most of these studies focus on their experimental substrates (and therefore rarely include samples from nearby sediments or natural rocks), understanding the possible mechanisms of this spillover effect remain limited by the incidental nature of the observations [13,23,25] and the understudied nature of these specialists’ behavior and lifecycles [26–28].

The overall objective of this research is to better understand the composition, resilience, and connectivity of deep-sea hard substrate communities by examining how macrofauna colonize different types of hard substrates, including organic fall and seep -associated substrates, under different oxygenation, depths, and proximities to shore. Wood and rocks were deployed in the SCB at two locations (inshore vs offshore), each with two different water depths (shallower vs deeper) to address the influence of substrate type, proximity to shore, and depth and oxygen concentration on the density, diversity, community composition, and role of specialist species in macrofaunal colonizers, including in comparison to natural, in situ assemblages. Experimental rock deployments included carbonate rocks, which are common at methane seeps found in the SCB, but which do not occur naturally at the experiment’s non-reducing deployment sites. These rocks were deployed with the objective of elucidating whether this rock type would attract specialist communities despite being deployed outside of a reducing ecosystem, or if its communities would remain similar to the communities found on other hard substrates that occur naturally at the deployment sites. We hypothesized that substrate type would influence both the density and community composition of macrofaunal colonizers, with substrates common in reducing ecosystems (wood, carbonate rock) exhibiting higher densities and different compositions than naturally occurring rocks. Density and diversity of macrofauna were expected to be higher closer to shore and where oxygen concentration was higher. Wood substrates were expected to exhibit high species dominance by wood-fall specialists. The wood borer *Xylophaga* was examined not only for its ability to modify habitat and influence wood associates, but also for its early life stage occurrence on non-wood substrates surrounding wood deployments in this experiment. The experimental juxtaposition of substrate type, depth/oxygen regime, and proximity to shore should inform the implications of disturbance from climate-induced deoxygenation and direct resource extraction.

## Methods

### Study area

**Fig 1:**
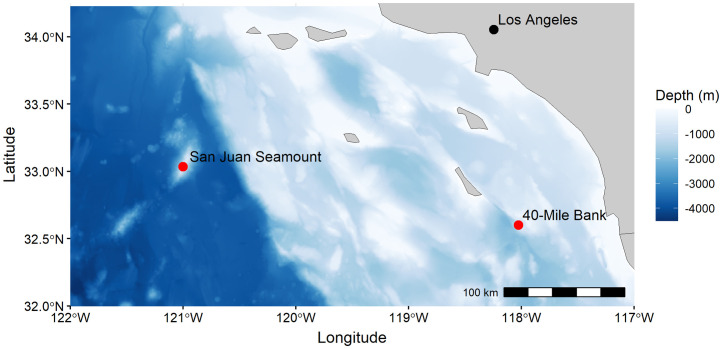
Deployment sites of the colonization experiment in the Southern California Borderland. Coastline and bathymetric data via open-access, public domain resources Natural Earth and NOAA National Bathymetric Source data, respectively.

The experiments took place on 40-Mile Bank (40MB; located approximately 70 km from shore) and San Juan Seamount (SJS; located approximately 200 km from shore) at bathyal depths in the Southern California Borderlands (SCB) (Fig 1). Each deployment area received experimental substrates in October/November 2020 at two different depths: ~ 700 m situated in the core of a well-developed Oxygen Minimum Zone (OMZ), representing lower oxygen exposures (7−9 μMol O_2_ L^-1^), and at ~1100 m along the lower boundary of the OMZ (22−23 μMol O_2_ L^-1^), representing higher oxygen exposures (Table 1). The design and deployment of this experiment was opportunistic, using substrates available on-board the *Nautilus* during the October/November 2020 voyage. Replicate deployments of the same substrate type were possible for wood and carbonate across all sites and depths, but not for the mineral-rich and sedimentary substrates (Table 1). Results related to the resulting small sample sizes and uneven array for certain substrate types are therefore interpreted with caution. No permits were required to complete this work because it did not involve threatened or endangered species or vertebrates, and was conducted in US federal waters outside State or MPA jurisdiction.

**Table 1:** Locations, environmental values (July 2021), and substrate types for experimental substrates collected 10 months after deployment and natural rocks (all collected in situ from deployment sites in July 2021) in the Southern California Borderland. Environmental values and associated samples were collected at San Juan Seamount on July 27, 2021 and at 40-Mile Bank on July 31, 2021.

Treatment	Site	Depth (m)	Temperature (°C)	Oxygen (µMol/L)	Latitude (ddeg)	Longitude (ddeg)	Substrates	n
Experimental	40-Mile Bank	692	5.65 ± 0.03	7.69 ± 0.03	32.59702	−118.017	Carbonate rock	2
							Ferromanganese rock	1
							Phosphorite rock	1
							Wood	2
	San Juan Seamount	694	4.95 ± 0.00	9.33 ± 0.03	33.03337	−121.000	Carbonate rock	2
							Sedimentary rock	1
							Wood	2
	40-Mile Bank	1039	3.91 ± 0.03	22.18 ± 0.49	32.59942	−118.025	Carbonate rock	2
							Ferromanganese rock	1
							Phosphorite rock	1
							Wood	2
	San Juan Seamount	1124	3.83 ± 0.02	22.72 ± 0.29	33.03954	−121.006	Carbonate rock	2
							Sedimentary rock	1
							Wood	2
Natural	40-Mile Bank	692	5.61 ± 0.01	7.53 ± 0.01	32.59740	−118.019	Basalt	3
							Sedimentary rock	1
	San Juan Seamount	692	4.96 ± 0.00	9.21 ± 0.00	33.03336	−121.000	Basalt	6
	40-Mile Bank	1039	3.96 ± 0.01	21.05 ± 0.26	32.59938	−118.025	Basalt	3
							Sedimentary rock	1
	San Juan Seamount	1124	3.74 ± 0.01	24.22 ± 0.12	33.03958	−121.006	Basalt	4
							Ferromanganese rock	3

The uneven array of substrate types across deployments was due to the opportunistic nature of this experiment. Standard errors of oxygen and temperature are extremely small due to low variability at sample sites; temperature values rounded to nearest 0.01°C for clarity.

### Experimental design

**Fig 2:**
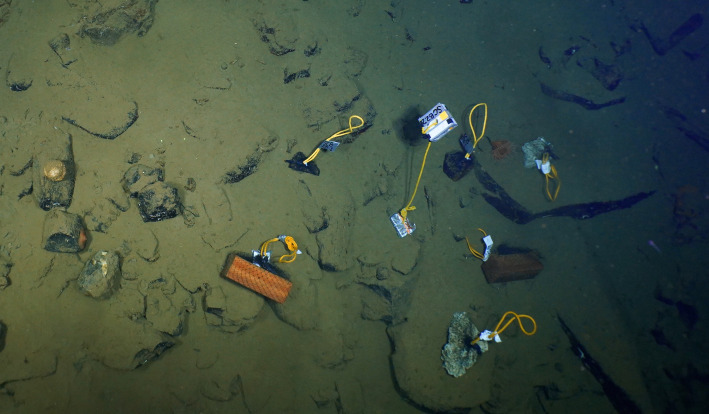
Deployed experimental hard substrates at 1039 m at 40-Mile Bank.

To address research objectives, defaunated rocks (surface area 323–1870 cm^2^) and untreated Douglas fir wood blocks (1075.62 cm^2^ [9.1x9.1x25 cm]) were deployed at each location and depth (4 total deployments) (Table 1). The substrates used were carbonate rocks previously collected from Costa Rica (2017–2019) then defaunated by removing visible fauna and air drying for 1 or more years. The ferromanganese, phosphorite, and sedimentary rocks were collected from the SCB in July 2020 and defaunated by removing visible fauna (both before and after sitting overnight in room-temperature water) and drying for several days before being deployed. To facilitate deployment and recovery, experimental substrates were individually wrapped in thin black plastic garden netting (mesh opening 1.2 cm) and affixed to polypro nylon rope loops with duct tape to allow for handling by ROV manipulators (Fig 2). Wood blocks were soaked in filtered seawater for 48 hours before deployment and duct tape-covered four-pound lead weights were affixed to the plastic mesh to ensure the wood blocks would remain stationary once deployed.

Each deployment set included two wood blocks, two carbonate rocks, and one or more other rocks (ferromanganese, sedimentary, or phosphorite rock) (Table 1; Fig 2). At each deployment site, experimental units were haphazardly placed in a roughly circular cluster, with individual substrates placed approximately 25–50 cm apart (Fig 2). Deployment occurred October 30, 2020 (SJS) and November 3, 2020 (40MB) by the ROV *Hercules* during the E/V *Nautilus* 2020 Southern California Borderland cruise (NA124). The experimental substrates remained at the four sites for approximately 10 months and were collected on July 27, 2021 (SJS) and July 31, 2021 (40MB) during the Schmidt Ocean Institute’s 2021 Biodiverse Borderlands Expedition on board the R/V *Falkor* (FK210726) using the ROV *SuBastian*. Experimental substrates were collected using the polypro nylon rope loops attached to the plastic garden netting around each substrate. This allowed for easier manipulation and reduced risk of cross contamination between samples from direct contact with the ROV manipulators. Four to seven naturally-occurring, unmanipulated rocks, referred to here as “natural,” were collected within 10 m of each colonization experiment deployment site during the deployment (October 30 and November 3, 2020) and recovery (July 27 and 31, 2021) dives to provide information about surrounding assemblages at the start and end of the experiment. During recovery, each experimental unit or natural rock was placed individually in one of four isolated plexiglass biobox inserts within a large delrin biobox to avoid contamination between samples. Extreme caution was taken to avoid possible contamination between biobox insert compartments during the recovery of substrates. However, it remains possible that fauna falling from the surface of a substrate during transport by the manipulator into the collection compartment could drift into a neighboring compartment within the same biobox before the biobox lid is closed. Once closed, the bioboxes are watertight and cross contamination between insert compartments is not possible. Natural rock substrate type (basalt, ferromanganese, or sedimentary) was identified after collection and processing as in Guraieb et al. [9] (Table 1).

### Sample processing

Upon recovery, experimental units were refrigerated at 4–6°C then photographed on six sides with a scale and label. All large, visible fauna were then removed using forceps. Rock surface area was measured by wrapping the substrate in a monolayer of aluminum foil, ensuring the foil was molded to the rock’s various features of surface texture (e.g., convex and concave shapes). The foil was then dried and weighed, and the surface area calculated using the known weight per cm^2^ of aluminum foil (as in Marsh, 1970 [29]). Wood blocks were initially 1075.62 cm^2^ in surface area; this value was used to estimate densities, although surface area generally increased in wood heavily bored by *Xylophaga*. The residue water contained in each biobox compartment was washed through a 300-micrometer mesh to collect macrofauna that had fallen off during recovery. Hard substrates were left in buckets of room-temperature seawater overnight to allow the remaining fauna to fall or crawl out of the crevices. The substrates and the residue water in the buckets were then also washed through a 300-micrometer mesh to recover macrofauna. All macrofauna sorted upon recovery or retained on the 300-micrometer mesh were combined and preserved in 70% ethanol for later sorting and identification and are the subject of this study. In the laboratory, each sample was washed over a 300-micrometer mesh sieve in freshwater and collected in petri dishes to be analyzed under a dissecting microscope at 25x magnification. Each animal was identified to morphospecies, or the lowest taxonomic level feasible.

**Fig 3:**
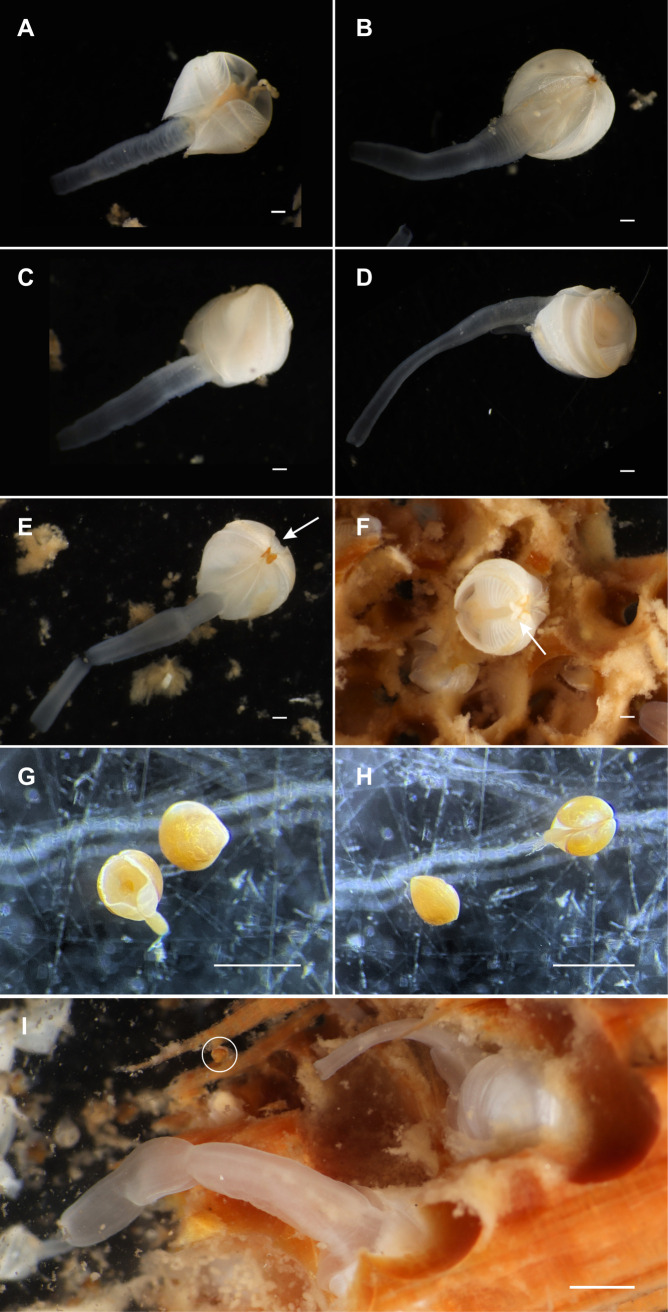
Images of adult and juvenile *Xylophaga* specimens from experimental wood substrates. **(A-F)** Images of live, post-metamorphosis *Xylophaga* specimens, classified as adults for the purpose of this study. Arrows indicate the triangular mesoplax characteristic of *X. washingtona*. Scale bars indicate 1 mm. **(G-H)** Images of small, pre-metamorphosis or partially-metamorphosized preserved *Xylophaga*, classified as juveniles for the purpose of this study. Scale bars indicate 1 mm. **(I)** Image of live *Xylophaga* inside of burrows on heavily bored wood substrate. Circle indicates a live juvenile *Xylophaga*. Scale bar indicates 1 cm. Images A-F and I courtesy of Greg Rouse.

To estimate adult *Xylophaga* counts, wood blocks were sliced into one-inch sections and all visible boreholes on the interior wall of four randomly selected slices were then counted and averaged. The total number of boreholes on each end of the wood blocks were also counted. The average number of boreholes counted per wood slice face was then multiplied by the length of the wood blocks (250 mm) divided by the average depth of the boreholes across the selected slices of wood (3 mm), and added to the total number of boreholes on the ends of the wood blocks to achieve an estimated number of adult *Xylophaga* across the whole block of wood. The exception to this method was for one of the wood samples from SJS at 694 m (SCB-058), due to the wood splitting lengthwise during processing. One side of the block was treated as the subsample wood slice for this sample and boreholes were counted via the same process described above, but multiplied by width (91 mm) rather than length in the calculation. Manual total counts of juvenile *Xylophaga* were made for all the rock samples and the two wood samples from San Juan Seamount at 694 m. The remaining wood samples were randomly split four times with a plankton splitter and were counted in one-sixteenth of the original sample under a dissecting microscope at 12x magnification. Only individuals with shells containing tissue were counted. Subsample counts were multiplied by 16 to estimate total juvenile *Xylophaga.* Juvenile *Xylophaga* were distinguished from adult *Xylophaga* morphologically, in line with descriptions of larval development for other members of the genus [25,27]: small (300–400 μm), orange, pre-metamorphosis or partially-metamorphosized individuals were classified as juveniles for the purpose of this study, while larger (2–3 mm), white, post-metamorphosis individuals were classified as adults (Fig 3).

Adult xylophagaid bivalves from the experimentally deployed wood were identified by sequencing the nuclear ribosomal RNA 18S subunit (18S) and the nuclear histone H3 gene (H3) as in Hiley et al. (2024) [30]. Five representative individuals were chosen for DNA extraction (at least one from each deployment location), and ethanol-fixed vouchers were deposited in the Scripps Institution of Oceanography Benthic Invertebrate Collection (SIO-BIC, catalog numbers M18423, M18424, M18426, M18427, M18429). DNA sequences were deposited in GenBank (accession numbers: 18S: PX310512, PX310513; H3: PX317690, PX317691, PX317692, PX317693, PX317694) and queried against other GenBank sequences using the nucleotide BLAST suite (https://blast.ncbi.nlm.nih.gov/) [31–33]. The H3 sequences of all five specimens were identical, indicating that they represent the same xylophagaid species. Two representatives, SIO-BIC M18427 (San Juan Seamount, experimental wood, 1124 m) and M18429 (San Juan Seamount, experimental wood, 694 m), were selected for sequencing of 18S, which is a commonly used species delimitation marker in Xylophagaidae [34–36]. The 18S sequences were 100% identical to a vouchered reference sequence of *Xylophaga washingtona* (JF899227.1) [34] and all other BLAST hits had ≤ 95% identity.

The morphological features of these specimens were also consistent with morphological descriptions of *X. washingtona* [37–39]. Adults had a globose, equivalve shell with a small triangular mesoplax (anterior to and between the umbos)(Fig 3E, F), and fine concentric ribs along the shell’s medial section, as well as an excurrent siphon of variable length (Fig 3A-E). The species has been reported from the Gulf of Alaska to southern California at 18–2,066 m depth [40], which encompasses our sampling localities [37]. As a result, the adult *Xylophaga* observed in this experiment are referred to as *X. washingtona*. Though it is likely that the observed juveniles are of the same species, because these specimens were not sequenced, they are referred to as “juvenile *Xylophaga*” for the purposes of this study.

### Statistical analyses

To assess the influence of temporal changes to macrofaunal communities between the beginning and end of the experiment, including as a result of the experiment itself, the community structure (density, diversity, and community composition) of 2020 natural rocks were compared to 2021 natural rocks. Counts for all identified taxa were standardized by the surface area of the substrates and represented as density per 200 cm^2^ (as in [9,14,15,41]) for plotting and statistical analysis. Even under log transformation, the distributions of macrofaunal densities were not normal and had non-homogeneous variance. As a result, non-parametric Wilcoxon (for two factor comparisons) and Kruskal-Wallis tests (for > 2 factor comparisons) were performed to calculate the significance of variability in macrofaunal densities and diversities across substrates, sites, depths, and oxygen levels. For significant Kruskal-Wallis tests, Dunn’s tests using the Benjamini-Hochberg adjustment [42] were performed. To maximize the interpretability of the diversity results, Hill numbers, including q = 0 (species richness, S), q = 1 (exponential of the Shannon Diversity Index, or effective species number, exp H’_*[loge]*_), and q = 2 (inverse Simpson, 1/D), are reported, in addition to Pielou’s evenness (J’), rarefaction (ES_(20)_), and taxonomic distinctness (∆).

Because each site had just two depth deployments to assess the influence of both depth and oxygen concentration, comparing between depth categories (shallow vs. deep) and oxygen concentration categories (lower vs. higher) resulted in identical comparisons. As a result, depth and oxygen are presented together as a single factor variable in the analysis.

Multivariate community analysis was conducted using PRIMER-e v8 and PERMANOVA+ [43,44] and using the standardized density data. The standardized density data were fourth-root transformed to reduce the influence of highly abundant species (i.e., *Xylophaga*) while maintaining variation among low- and moderate-abundance species. This transformation is commonly applied in ecological community analyses (e.g., [45]) and is particularly suitable for datasets like this one that contain a mixture of rare species (including zeros), moderately abundant species, and a few highly abundant species, as it reduces the influence of highly abundant species while avoiding the compression of moderate abundances (e.g., under log transformation). Bray-Curtis dissimilarity matrices were then calculated to conduct permutational multivariate analysis of variance (PERMANOVA) and similarity percentages (SIMPER) analyses, and to prepare data visualization plots (e.g., multidimensional scaling plots). PERMANOVA tests were run for each dataset using relevant substrate and environmental factors. For PERMANOVA results, degrees of freedom (df) correspond to the numerator (factor) and denominator (residual) values used in the pseudo-F calculation; some denominator df values may be non-integer due to unbalanced designs. SIMPER analyses were run on these same datasets to assess the contribution of individual taxa to the PERMANOVA results. Canonical analysis of principal components (CAP) was used to visualize significant community effects on experimental substrates by providing a constrained ordination that maximized differences among *a priori* groups [46].

Linear regression on log-transformed data was used to assess relationships between *Xylophaga* abundance and that of certain wood-specialist polychaete taxa. Given the small sample size (n = 8), simple log–log linear regression was preferred over nonlinear models, which failed to converge or yielded unstable estimates. Although residuals showed minor deviations from normality, permutation tests confirmed the significance of slope estimates, indicating that the linear regression results are robust.

As adult *Xylophaga washingtona* were present on wood substrates in high abundances but absent from all other experimental and natural substrates collected, separate calculations were performed to analyze the significance of variability in densities of taxonomic groups across factors without the high densities of adult or juvenile *Xylophaga* masking trends in the densities of non-xylophagaid fauna from the phylum Mollusca. All calculations were done inclusive of *Xylophaga* unless reported otherwise. Where the exclusion of *Xylophaga* resulted in a change in statistical significance of a univariate or multivariate test, the results are stated separately.

## Results

### Density

The average density of colonizing macrofauna on experimental substrates was highest on wood (2,493 ± 657 indiv./200 cm^2^), followed by ferromanganese rock (132 ± 7 indiv./200 cm^2^), carbonate rock (59 ± 25 indiv./200 cm^2^), sedimentary rock (40 ± 34 indiv./200 cm^2^), and phosphorite rock (39 ± 0 indiv./200 cm^2^). When densities were compared by substrate type (across deployments), density differed significantly only between wood and carbonate rock (z = −3.080, p = 0.021), though the lack of significant difference between wood and other experimental rock substrates may have been due to the experiment’s limited sample sizes for those substrates. There was no significant difference in density among experimental rock types. Total colonizer densities did not vary significantly when compared by proximity to shore (40MB vs SJS: W = 33, p = 0.080) or water depth/oxygen (shallow vs deep; $\chi_{\text{3}}^{\text{2}}$= 5.758, p = 0.124), indicating that substrate type played a stronger role in density trends than environmental factors.

Densities of non-xylophagaid invertebrates (wood: 82 ± 27 indiv/200 cm^2^; ferromanganese rock: 50 ± 19; phosphorite rock: 19 ± 0; carbonate rock: 18 ± 5; sedimentary rock: 9 ± 5) did not exhibit statistical differences when comparing substrate type (across deployments; $\chi_{\text{4}}^{\text{2}}$ = 6.407, p = 0.171), indicating the outsized role *Xylophaga* had on determining substrate-specific density trends. Without *Xylophaga*, environmental factors played a more significant role in explaining variation in non-xylophagaid density patterns. Densities were significantly higher inshore (40MB, both depths) than offshore (SJS, both depths; W = 23, p = 0.016) by nearly a factor of two (Table 2). However, densities still did not differ significantly between water depths at 40MB (40MB 1039 m vs. 692 m: z = 0.800, p = 0.508) or SJS (SJS 1124 m vs. 694 m: z = 2.046, p = 0.082).

**Table 2:** Average densities of macrofauna (individuals/200 cm^2^) on experimental and natural substrates, by deployment site.

Treatment	Substrate	n	Site	Depth (m)	Annelida density	Arthropoda density	Mollusca (non-*Xylo.*) density	Adult *Xylophaga* density	Juvenile *Xylophaga* density	Echinodermata density	Cnidaria density	Other density	Total density	Total (non-*Xylo*.) density
Experimental	Ferromanganese Rock	1	40-Mile Bank	691	4.49 ± 0	20.21 ± 0	0 ± 0	0 ± 0	94.88 ± 0	4.49 ± 0	0 ± 0	1.12 ± 0	125.19 ± 0	30.32 ± 0
	Carbonate Rock	2	40-Mile Bank	692	4.05 ± 1.43	4.8 ± 2.83	0.7 ± 0.26	0 ± 0	82.93 ± 72.01	6.5 ± 2.79	0 ± 0	0 ± 0	98.98 ± 79.32	16.05 ± 7.31
	Phosphorite Rock	1	40-Mile Bank	692	4.17 ± 0	1.6 ± 0	0 ± 0	0 ± 0	20.21 ± 0	12.83 ± 0	0 ± 0	0 ± 0	38.82 ± 0	18.61 ± 0
	Wood	2	40-Mile Bank	692	50.3 ± 16.46	10.13 ± 7.16	0 ± 0	1191.13 ± 87.21	1140.09 ± 177.85	0.19 ± 0.19	0.09 ± 0.09	0 ± 0	2391.92 ± 274.07	60.71 ± 9.02
	Carbonate Rock	2	San Juan Seamount	694	2.18 ± 1.18	5.53 ± 3.92	0 ± 0	0 ± 0	0.41 ± 0.2	1.04 ± 0.43	0 ± 0	0.51 ± 0.09	9.66 ± 5.24	9.25 ± 5.44
	Sedimentary Rock	1	San Juan Seamount	694	0.74 ± 0	1.49 ± 0	0 ± 0	0 ± 0	1.49 ± 0	1.49 ± 0	0 ± 0	0 ± 0	5.21 ± 0	3.72 ± 0
	Wood	2	San Juan Seamount	694	1.12 ± 0.56	1.12 ± 0.19	0 ± 0	58.01 ± 23.24	8.65 ± 1.95	0.46 ± 0.09	0 ± 0	0 ± 0	69.36 ± 20.45	2.7 ± 0.84
	Carbonate Rock	2	40-Mile Bank	1039	10.79 ± 3.93	4.69 ± 0.9	0.07 ± 0.07	0 ± 0	70.21 ± 44.05	15.55 ± 9.96	0.28 ± 0.28	0.42 ± 0.42	102.01 ± 57.82	31.8 ± 13.77
	Ferromanganese Rock	1	40-Mile Bank	1039	12.43 ± 0	21.13 ± 0	1.86 ± 0	0 ± 0	70.21 ± 0	32.93 ± 0	0 ± 0	0.62 ± 0	139.18 ± 0	68.97 ± 0
	Phosphorite Rock	1	40-Mile Bank	1039	3.72 ± 0	2 ± 0	0 ± 0	0 ± 0	20.33 ± 0	12.89 ± 0	0 ± 0	0 ± 0	38.94 ± 0	18.61 ± 0
	Wood	2	40-Mile Bank	1039	145.4 ± 75.31	4 ± 1.21	0.37 ± 0.37	672.08 ± 56.25	3590.86 ± 44.63	4.09 ± 1.49	0 ± 0	0.19 ± 0.19	4416.99 ± 25.1	154.05 ± 75.77
	Carbonate Rock	2	San Juan Seamount	1124	6.54 ± 1.54	3.85 ± 0.39	0.87 ± 0.1	0 ± 0	9.8 ± 9.8	2.21 ± 0.67	0 ± 0	0.38 ± 0.38	23.65 ± 6.71	13.85 ± 3.09
	Sedimentary Rock	1	San Juan Seamount	1124	5.29 ± 0	1.06 ± 0	1.06 ± 0	0 ± 0	60.3 ± 0	5.29 ± 0	0 ± 0	1.06 ± 0	74.05 ± 0	13.75 ± 0
	Wood	2	San Juan Seamount	1124	105.8 ± 40.35	3.07 ± 2.14	0.19 ± 0.19	1045.91 ± 135.55	1938.23 ± 1259.92	0.65 ± 0.46	0 ± 0	0 ± 0	3093.84 ± 1437.31	109.7 ± 41.84
Natural 2021	Basalt	3	40-Mile Bank	692	2.82 ± 0.29	2.83 ± 0.53	0.32 ± 0.11	0 ± 0	20.82 ± 10.40	4.35 ± 1.20	0.26 ± 0.11	0.05 ± 0.03	31.67 ± 12.39	10.85 ± 2.01
	Sedimentary rock	1	40-Mile Bank	692	1.18 ± 0	0.64 ± 0	0.11 ± 0	0 ± 0	1.18 ± 0	2.25 ± 0	0 ± 0	0 ± 0	5.35 ± 0	4.17 ± 0
	Basalt	6	San Juan Seamount	692	0.94 ± 0.11	0.81 ± 0.12	0.23 ± 0.05	0 ± 0	1.32 ± 0.40	1.43 ± 0.12	0.53 ± 0.12	0.35 ± 0.03	5.69 ± 0.68	4.37 ± 0.32
	Basalt	2	40-Mile Bank	1039	1.23 ± 0.20	0.93 ± 0.12	0.54 ± 0.16	0 ± 0	21.39 ± 5.45	1.43 ± 0.65	0.08 ± 0.06	0.08 ± 0.06	18.93 ± 4.86	3.59 ± 0.53
	Sedimentary rock	1	40-Mile Bank	1039	1.22 ± 0	0 ± 0	0.81 ± 0	0 ± 0	3.24 ± 0	0 ± 0	0 ± 0	0 ± 0	5.27 ± 0	2.03 ± 0
	Ferromanganese Rock	3	San Juan Seamount	1122	1.57 ± 0.46	1.23 ± 0.46	0.44 ± 0.06	0 ± 0	0.28 ± 0.12	1.00 ± 0.21	0.22 ± 0.06	0.17 ± 0.06	4.91 ± 0.80	4.63 ± 0.69
	Basalt	4	San Juan Seamount	1124	1.16 ± 0.22	0.88 ± 0.10	0.71 ± 0.20	0 ± 0	3.04 ± 0.87	1.09 ± 0.06	0 ± 0	0.17 ± 0.05	7.10 ± 0.88	4.07 ± 0.23

**Fig 4:**
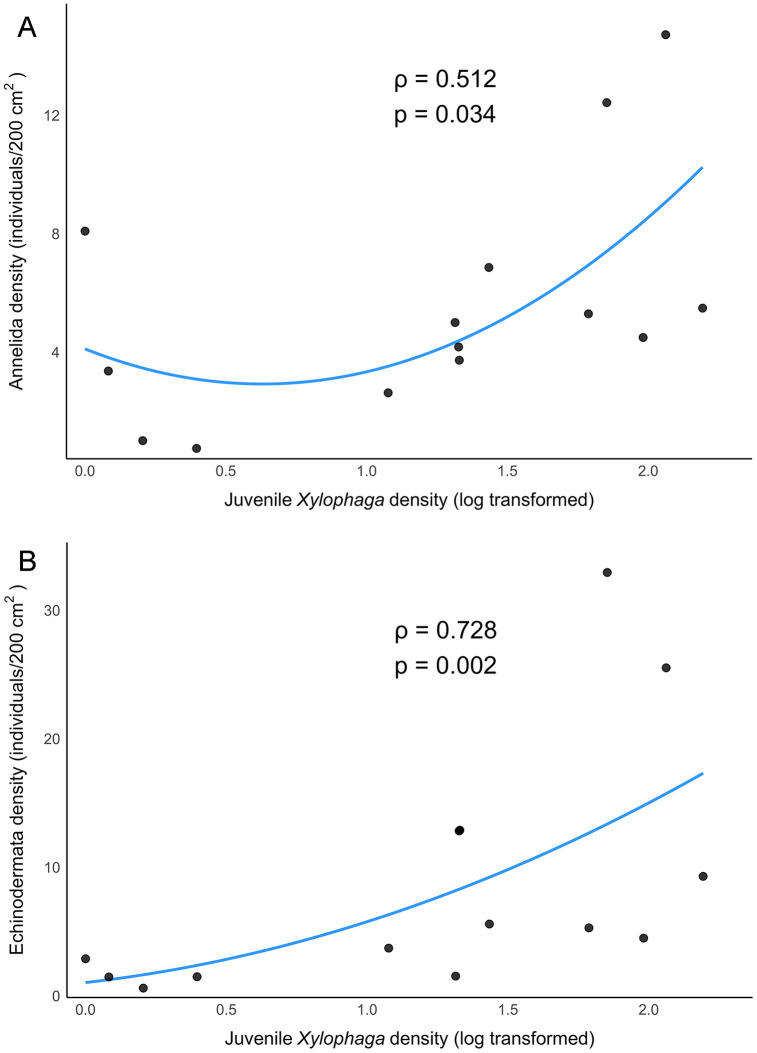
Scatter plot of juvenile *Xylophaga* densities and associated taxa on experimental rock substrates. **(A)** Plot of juvenile *Xylophaga* and annelid densities. **(B)** Plot of juvenile *Xylophaga* and echinoderm densities. The Spearman’s correlation coefficient, rho (ρ), and the significance of the correlation (p at α = 0.05) between *Xylophaga* and each taxon are given for each associated figure.

Juvenile *Xylophaga* were found on nearly all experimental substrates, including the rock substrates, and on most of the natural rocks collected in 2021 (Table 2). Upon recovery from the bioboxes, all substrates were washed to remove attached fauna. As a result, it is unknown how firmly attached the juvenile *Xylophaga* were to the rocks, or if they were attempting to bore into the rocks. No xylophagaid bivalves of any life stage were found on natural rocks collected in 2020 and no natural wood was observed in the vicinity. No adult or other later developmental stages of juvenile *X. washingtona* were found on any rock substrates. Juvenile *Xylophaga* densities were significantly, positively correlated with annelid (Spearman’s ρ = 0.512, p = 0.034) and echinoderm (ρ = 0.728, p = 0.002) densities on experimental rocks (combined deployment data; Fig 4), but not on 2021 natural rocks.

Adult *X. washingtona* densities differed significantly among substrate types (combined deployment data; $\chi_{\text{4}}^{\text{2}}$= 19.66, p < 0.001); adults were exclusively found on wood substrates (Table 2). Densities of adult *X. washingtona* ranged from 35 to 1278 individuals/200 cm^2^, depending on site and depth. Average density of juvenile *Xylophaga* found on wood substrates was more than an order of magnitude higher than those found on any other substrate type (Table 2). However, juvenile *Xylophaga* densities did not differ significantly across substrate types ($\chi_{\text{4}}^{\text{2}}$ = 7.296, p-value = 0.121), nor did any phylum exhibit significant differences in density across substrate types (combined deployment data). However, this result may have been influenced by the unexpectedly low *Xylophaga* densities at the shallow SJS deployment which, though not statistically significant, exhibited much lower densities of adult and juvenile *Xylophaga* than other wood substrate deployments (Table 2). The shallow (692 m) 40MB deployment exhibited significantly higher densities of juvenile *Xylophaga* (over 40x on average) than the shallow (694 m) SJS deployment (z = 2.501, df = 1, p = 0.037). *Xylophaga* juveniles were absent only on four rocks, all of which were sampled offshore (at SJS). The lack of juveniles on these rocks may have been due to the rocks’ distance from the colonization experiment. Three of the four rocks found without juvenile *Xylophaga* were natural rocks, which were collected further away from the experimental deployments at both shallow and deep deployments. Also, the lower densities of *Xylophaga* observed at the SJS wood deployments may have resulted in reduced “spillover” onto nearby rocks; all four of the rocks without juvenile *Xylophaga*––two natural and two experimental rocks––were collected from the offshore SJS sites (S1 Table).

Three groups exhibited greater densities on experimental substrates closer to shore (40MB, both depths) than offshore at SJS (both depths): arthropods (W = 27.5, p-value = 0.035), juvenile *Xylophaga* (W = 23, p-value = 0.014), and echinoderms (W = 22.5, p-value = 0.015; Table 2). When both depth and site were considered simultaneously for their impact on the densities of different macrofaunal groups, both annelid ($\chi_{\text{3}}^{\text{2}}$= 11.741, p = 0.008) and juvenile *Xylophaga* ($\chi_{\text{3}}^{\text{2}}$= 9.537, p = 0.023) densities were found to differ significantly. Annelid densities were significantly higher at the deeper depth (1124 m) than the shallow depth (694 m) at SJS (z = 2.825, df = 1, p = 0.014), and significantly higher at 40MB’s shallow (692 m) deployment than at SJS’s shallow (694 m) deployment (W = 1, p = 0.009), both of which may be related to the lower *Xylophaga* densities at the shallow SJS location discussed above (Table 2). Additionally, the deep (1039 m) 40MB deployment exhibited higher densities of echinoderms than the deep (1124 m) SJS deployment (W = 2, p = 0.017; Table 2), suggesting the broader influence of proximity to shore.

**Fig 5:**
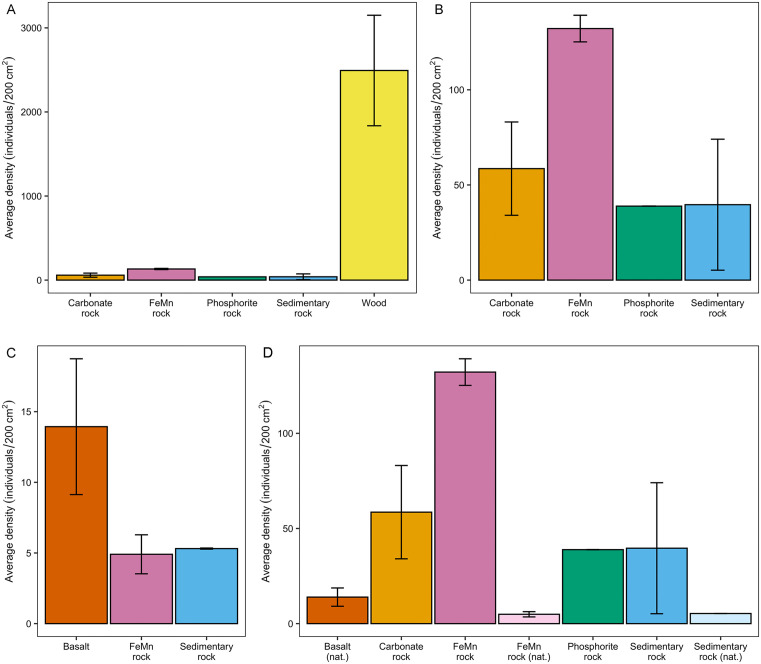
Average densities of macrofauna found on experimental and natural substrates. Plots show average macrofauna densities on (A) all experimental substrates, (B) experimental substrates excluding wood, (C) 2021 natural rocks, and (D) all substrates collected in 2021 (natural rocks denoted by nat.). Error bars indicate ± 1 standard error.

Colonizer densities on experimental substrates ranged from 4 to 4532 individuals/200 cm^2^ while densities on the 2021 natural (non-experimental) rocks ranged from 2 to 74 individuals/200 cm^2^ (Fig 5). Macrofaunal densities on 2021 natural rocks did not vary significantly across substrate types (combined deployment data; basalt vs. ferromanganese vs. sedimentary rock: $\chi_{\text{3}}^{\text{2}}$= 1.169, p-value = 0.558). Macrofaunal densities on experimental substrates were approximately 80x greater than on the 2021 natural rocks (combined deployment data; W = 44.5, p < 0.001). Across substrates and based on data combined across deployments, densities were significantly higher on experimental wood substrates than on 2021 natural ferromanganese rocks (z = −3.461, df = 1, p < 0.008) and on 2021 natural basalt rocks (z = −4.379, df = 1, p < 0.001). Juvenile *Xylophaga* densities were significantly greater on 2021 natural rocks (z = −2.723, df = 1, p = 0.007) than in 2020 (none were present in 2020). However, non-xylophagaid total macrofaunal densities were not significantly different between 2021 and 2020 natural rocks (W = 94, p = 0.81).

### Diversity

Among experimental substrates, average diversity was highest on carbonate rocks (combined deployment data; avg. q = 1, or exp H’_*[loge]*_ = 7.55 ± 1.88; avg. q = 2, or 1/D: 5.29 ± 1.53; avg. ES_(20)_ = 7.88 ± 1.30; avg. Δ = 62.00 ± 7.31; n = 8) and lowest on wood (combined deployment data; avg. q = 1, or exp H’_*[loge]*_ = 2.12 ± 0.13; avg. q = 2, or 1/D: 1.75 ± 0.12; avg. ES_(20)_ = 2.65 ± 0.03; avg. Δ = 9.09 ± 1.26; n = 8). Sedimentary rock exhibited the highest average evenness (combined deployment data; avg. J’ = 0.66 ± 0.32, n = 2), followed closely by carbonate rock (combined deployment data; avg. J’ = 0.63 ± 0.09, n = 8), while wood exhibited the lowest average evenness (combined deployment data; avg. J’ = 0.27 ± 0.03, n = 8). Richness was highest on ferromanganese rocks (combined deployment data: avg. q = 0, or S = 24.00 ± 8.00, n = 2) and lowest on sedimentary rock (combined deployment data: avg. q = 0, or S = 10.00 ± 4.00, n = 2). Macrofaunal diversity (q = 1, or exp H’_*[loge]*_) differed significantly among experimental substrate types (combined deployment data; Kruskal-Wallis H = 11.46, df = 4, p = 0.005), however, there was no significant difference in richness (q = 0: H = 4.35, df = 4, p = 0.39) or diversity at q = 2 (1/D; H = 6.63, df = 4, p = 0.13), indicating the substrates’ communities differed most in their relative abundances of taxa with intermediate densities, rather than the number of species or the dominant community structure. There was a significant difference between natural and experimental substrates (combined deployment data, across all substrates) at q = 1 (exp H’_*[loge]*_; H = 9.52, df = 1, p = 0.0017) and q = 2 (1/D; H = 7.47, df = 1, p = 0.0045), but not at q = 0 (S; H = 1.83, df = 1, p = 0.17), which matches observed trends of dominance by wood specialists on experimental substrates. Macrofaunal diversity did not differ significantly among the natural rock substrates from 2021 (natural basalts, ferromanganese, and sedimentary rocks from combined deployment data) for any of the calculated diversity metrics.

Richness (q = 0: H = 9.93, df = 1, p < 0.001), diversity (q = 1: H = 8.04, df = 1, p = 0.003; q = 2: H = 5.34, df = 1, p = 0.021; ES_(20)_: H = 10.60, df = 1, p < 0.001), evenness (J’: H = 6.89, df = 1, p = 0.008), and taxonomic distinctness (∆: H = 11.29, df = 1, p < 0.001) were significantly higher on experimental carbonate rocks than on wood across deployments, but small sample sizes inhibited robust testing of differences in diversity among the other experimental substrates (i.e., sedimentary, phosphorite, and ferromanganese rocks) (Table 3). Across deployment locations, natural rocks exhibited significantly higher diversity (q = 1: H = 9.52, df = 1, p = 0.001; q = 2: H = 7.47, df = 1, p = 0.0045; ES_(20)_: H = 12.79, df = 1, p < 0.001), evenness (J’: H = 12.88, df = 1, p < 0.001), and taxonomic distinctness (∆: H = 11.73, df = 1, p < 0.001) than experimental substrates, but not higher richness (q = 0: H = 1.83, df = 1, p = 0.18) (Table 3). Colonizer richness (q = 0), diversity (q = 1 and 2), evenness, and taxonomic distinctness did not differ significantly between sites (combined depths). Colonizer richness (q = 0: H = 5.96, df = 1, p = 0.012) and diversity at q = 1 (H = 4.28, df = 1, p = 0.041) were higher at deeper depths (combined sites), but evenness, diversity at q = 2 and taxonomic distinctness did not differ significantly between the two depths. Natural rocks exhibited higher taxonomic distinctness at SJS than 40MB (combined depths; ∆: H = 4.75, df = 1, p = 0.03), but showed no significant difference in other diversity metrics between sites. Natural rock richness (q = 0; H = 3.73, df = 1, p = 0.046) and diversity (q = 1: H = 5.23, df = 1, p = 0.020; and q = 2: H = 6.24, df = 1, p = 0.011) were higher at shallower depths (combined sites).

**Table 3:** Average diversity values ± 1 standard error for experimental substrates and natural rocks collected in 2021 according to substrate type.

Treatment	Substrate	S, Hill q = 0	exp(H’_*[loge]*_), Hill q = 1	1/D, Hill q = 2	ES_(20)_	J’	∆
Experimental	Wood	16.63 ± 2.04	2.12 ± 0.13	1.75 ± 0.12	2.65 ± 0.13	0.27 ± 0.03	9.09 ± 1.3
	Carbonate Rock	20.50 ± 3.46	7.55 ± 1.88	5.29 ± 1.53	7.88 ± 1.30	0.63 ± 0.09	62.00 ± 7.31
	Sedimentary Rock	10.00 ± 4.00	4.09 ± 1.65	3.47 ± 1.97	4.91 ± 1.08	0.66 ± 0.32	56.02 ± 23.4
	Ferromanganese Rock	24.00 ± 8.00	4.24 ± 1.39	2.40 ± 0.70	5.26 ± 0.83	0.44 ± 0.06	53.94 ± 13.0
	Phosphorite Rock	13.50 ± 0.50	4.15 ± 0.32	2.78 ± 0.17	4.85 ± 0.55	0.55 ± 0.04	96.06 ± 3.21
	*Average*	16.93 ± 1.11	4.65 ± 0.84	3.35 ± 0.65	5.11 ± 0.37	0.51 ± 0.03	48.55 ± 6.20
	*Average (rocks)*	17.00 ± 1.60	6.10 ± 1.09	4.26 ± 0.88	5.73 ± 0.36	0.57 ± 0.02	58.42 ± 4.92
Natural (2021)	Basalt	14.47 ± 1.56	8.17 ± 1.13	6.19 ± 1.01	9.37 ± 1.03	0.76 ± 0.06	69.12 ± 5.30
	Sedimentary Rock	12.00 ± 6.00	7.13 ± 3.51	4.64 ± 2.19	6.45 ± 0.55	0.77 ± 0.05	69.16 ± 13.3
	Ferromanganese Rock	14.00 ± 4.36	11.91 ± 3.39	9.82 ± 2.53	12.66 ± 3.67	0.95 ± 0.01	89.14 ± 2.00
	*Average*	13.49 ± 0.44	8.62 ± 1.03	6.58 ± 0.89	9.49 ± 1.04	0.83 ± 0.04	75.81 ± 3.85

Average diversity values above include: species richness (S or Hill q = 0), exponential Shannon entropy (exp H’_*[loge]*_ or q = 1), inverse Simpson index (1/D or q = 2), expected species richness for a standardized sample size of 20 individuals (ES_(20)_), Pielou’s evenness index (J’), and taxonomic distinctness (Δ).

### Community composition

**Fig 6:**
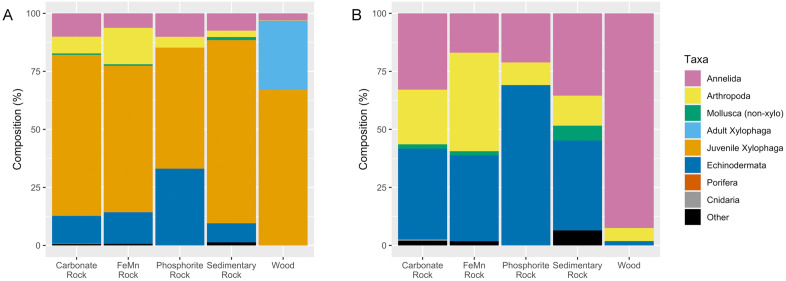
Community composition of experimental substrates according to substrate type, across deployments. Percent composition of the major taxa found on experimental substrates, by substrate type, both with (A) and without (B) the inclusion of *Xylophaga*.

**Fig 7:**
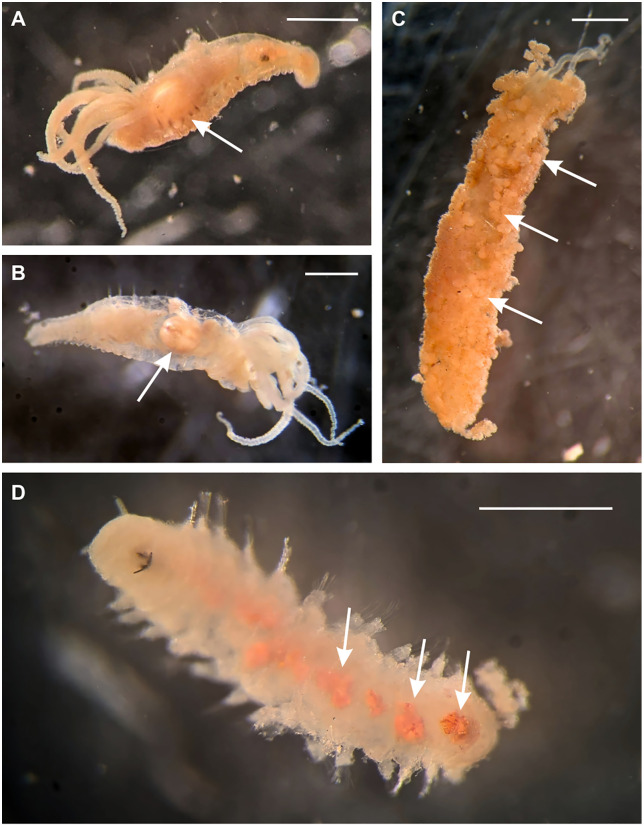
Observations of trophic connections between *Xylophaga* and annelids on experimental wood substrates. **(A-B)** Images of juvenile *Decemunciger sp.* individuals with juvenile *Xylophaga* inside their body cavities; (C) an adult *Decemunciger sp.* tube constructed with *X. washingtona* fecal pellets; (D) a dorvilleid individual with *X. washingtona* fecal pellets or *Decemunciger sp.* tube pieces inside its body cavity. Scale bars indicate 1 mm.

**Fig 8:**
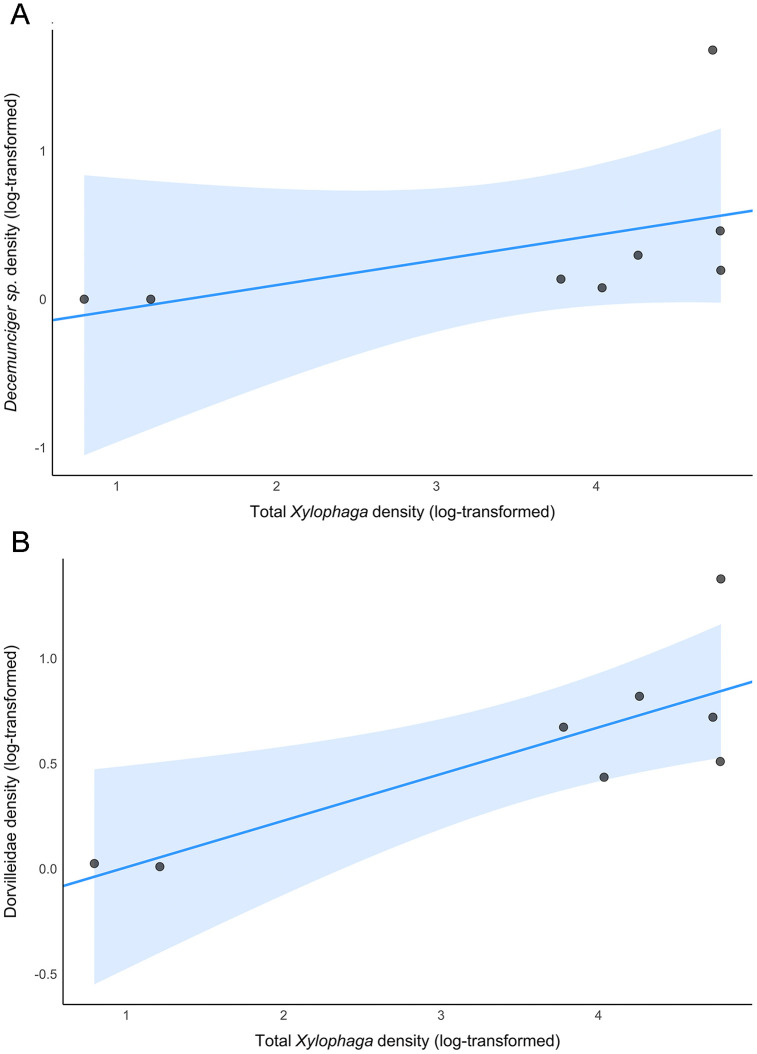
Linear regression plots depicting the relationships between total *Xylophaga* sp. densities and associated annelid densities on experimental wood substrates across deployments. Linear regression plots depicting (A) log-transformed *Decemunciger* sp. density (indiv./200 cm^2^) and (B) log-transformed Dorvilleidae density (indiv./200 cm^2^) as a function of log-transformed total *Xylophaga* density (combined adult *X. washingtona* and juvenile *Xylophaga* densities; indiv./200 cm^2^). The blue line represents the fitted regression line, while the shaded area indicates the 95% confidence interval for the regression line. Data points are represented by circles.

Wood substrates were dominated by adult *X. washingtona* and juvenile *Xylophaga*, which together accounted for 96.76% of individuals colonizing wood blocks across deployments. When *Xylophaga* were excluded, annelids dominated wood substrates (92.6% of non-xylophagaid individuals), while carbonate rocks exhibited a more even distribution among high-level taxa across deployments (Fig 6). In wood samples (combined deployment data), *Xylophaga* dominance was positively correlated with high densities of certain annelids (e.g., *Decemunciger* sp. and Dorvilleidae), some of which were observed to contain *X*. *washingtona* fecal pellets in their body cavities or in constructed tubes (Fig 7). Linear regression analysis on log-transformed values revealed a significant positive relationship between *Decemunciger* sp. abundance and total *Xylophaga* abundance (combined adult *X. washingtona* and juvenile *Xylophaga*) across deployments (F₁,₆ = 27.3, p = 0.002), with variations in *Xylophaga* abundance describing ~82% of the variation in *Decemunciger* sp. (R^2^ = 0.82, adjusted R² = 0.79). The same pattern was detected for Dorvilleidae polychaete abundance and total *Xylophaga* abundance across deployments (F₁,₆ = 51.2, p < 0.001), with variation in *Xylophaga* abundance describing ~90% of the variation in Dorvilleidae abundance (R² = 0.90, adjusted R² = 0.88) (Fig 8).

Experimental carbonate rock macrofaunal communities were also dominated by juvenile *Xylophaga* (70.93%; combined deployment data), but exhibited less dominance by *Xylophaga*-related annelids and greater compositional diversity (Fig 6), with only one *Decemunciger sp.* (Ampharetidae) individual found off wood, on one carbonate rock. Though dorvilleid polychaetes were consistently present on both wood and carbonate rocks, dorvilleid densities on carbonate rocks across deployments were approximately 10% of the densities found on wood substrates across deployments. No adult *X. washingtona* were found on experimental carbonate rocks or any other non-wood hard substrates. With all *Xylophaga* excluded, deep deployments at both 40MB and SJS were dominated by annelids (82.76% and 92.30% Annelida, respectively), although 40MB exhibited a higher proportion of echinoderms (12.09% Echinodermata at 40MB vs 1.87% at SJS). The shallow deployment at 40MB was also dominated by annelids (72.68%), but with a higher proportion of arthropods (19.21% Arthropoda at 40MB 692 m vs 4.3% at 40MB 1039 m). The shallow deployment at SJS had macrofaunal composition most dissimilar to the other sites, with highest dominance by arthropods (60.19%), followed by annelids (25.00%), echinoderms (10.19%), and others (4.63%). It bears noting that SJS at 694 m was also the deployment that exhibited the lowest total densities of *Xylophaga* by more than an order of magnitude (Table 2). As no known *Xylophaga* predators were observed on the wood at this location, environmental conditions remain the most likely explanation for this trend.

Within experimental substrates, community composition differed significantly among substrate types (combined deployment data; pseudo-F = 3.37, df = 4, p < 0.001) and between the two water depths (combined sites; pseudo-F = 3.79, df = 1, p = 0.006). The interactions between substrate and site (pseudo-F = 2.57, df = 1, p = 0.02) and between substrate and depth (pseudo-F = 1.63, df = 4, p = 0.03) were each significant. Among substrate types (combined deployments), pairwise tests indicated a significant difference in composition between wood and carbonate rock (t = 3.01, df = 8, p < 0.001), wood and phosphorite (t = 2.67, df = 2.48, p = 0.02), wood and ferromanganese rock (t = 2.61, df = 2.48, p = 0.02), and wood and sedimentary rock (t = 2.74, df = 2.48, p = 0.02). Community composition differed significantly in pairwise tests between sites (combined depths) for carbonate rock (t = 1.61, df = 4, p = 0.04) and wood (t = 2.46, df = 4, p = 0.03), as well as between carbonate rock and wood substrates at both SJS (t = 2.00, df = 4, p = 0.03) and 40MB (t = 2.92, df = 4, p = 0.03). Pairwise tests of the substrate-depth interaction revealed significant differences in community composition between deep and shallow deployments of wood (t = 2.78, df = 4, p = 0.03), and between wood and the other experimental substrates, across depths (Table 4). Though multiple taxa showed a strong preference for wood substrates over rock substrates (see below), there was no significant difference among communities on different experimental rock substrates (combined deployments), including between mineral-rich and carbonate rocks (pseudo-F = 1.22, df = 1, p = 0.33). Rock communities were dominated by singletons and doubletons, with only juvenile *Xylophaga* sp. and Cirripedia larvae (ecdysis stage) found across all substrate types. *Munnopsurus sp. a* (Isopoda) exhibited the highest abundances on carbonate rocks, but was also found on wood, ferromanganese rocks, and phosphorite rocks. While no individual species exhibited a strong preference for experimental rock substrates, some larger taxonomic groups (e.g., ophiuroids) did exhibit higher relative densities on rock substrates than wood. Additionally, one individual rock sample (the experimental ferromanganese rock at 40MB 1039 m) exhibited high densities of Gammaridae sp. b, which was otherwise found in high densities only on shallow (692 m) wood substrates at 40MB. Overall, most of the observed trends in community composition were driven by the distinct communities found on experimental wood substrates.

**Table 4:** Results of pairwise PERMANOVA analysis for the interaction between substrate type and depth.

Depth	Substrate pairing	t	Den. df*	p	Perms**
Shallow	Wood vs carbonate rock	2.329	4	0.031	270
	Wood vs phosphorite rock	3.084	2	0.035	30
	Wood vs ferromanganese rock	2.855	2	0.032	30
	Wood vs sedimentary rock	3.122	2	0.036	30
Deep	Wood vs carbonate rock	2.283	4	0.031	270
	Wood vs phosphorite rock	3.092	2	0.036	30
	Wood vs ferromanganese rock	3.153	2	0.034	30
	Wood vs sedimentary rock	3.245	2	0.033	30

*Den. df indicates the denominator (residual) degrees of freedom from permutation tests. **Perms indicates unique permutations used by the analysis. Differences in unique permutations were due to limitations caused by the small sample sizes of the experiment. Results with low permutations were interpreted with caution.

**Fig 9:**
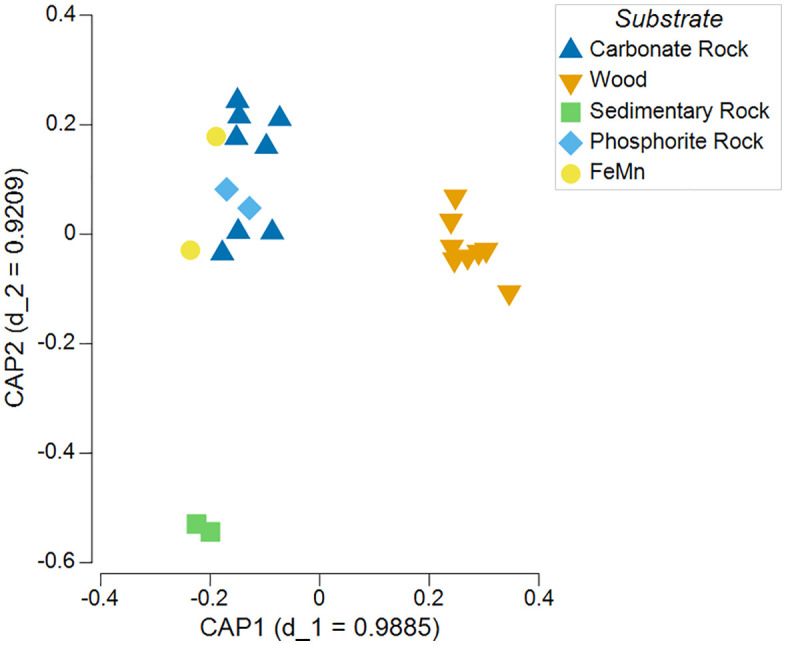
CAP plot of macrofaunal colonizers on experimental substrates. Constrained ordination plot via canonical analysis of principal components (CAP) of *a priori* experimental substrate groups showing significant grouping among carbonate rock and wood substrates. Substrate comparison based on combined deployment data.

The taxa contributing most to the dissimilarity between communities colonizing carbonate rock and wood substrates across deployments were adult *X*. *washingtona* (10.94%), followed by juvenile *Xylophaga* sp. (8.10%), *Ophryotrocha* sp. *2* (3.06%), *Decemunciger* spp. (2.92%), and unid. dorvelleid (2.69%; SIMPER, average dissimilarity = 81.54), all of which were more abundant on wood. Across deployments, wood samples exhibited greater average within-substrate similarity (SIMPER 50.29) than carbonate (SIMPER 22.92), ferromanganese (SIMPER 22.78), phosphorite (SIMPER 20.94), or sedimentary rock samples (SIMPER 10.52). However, this is largely a result of the dominance of *Xylophaga* (both adult and juvenile) in the wood samples, which contributed a cumulative percent contribution of 57.84% to within-group similarity for wood samples (SIMPER). Only wood and carbonate rock substrates had sufficient replication to reveal substrate preferences, with strong, significant grouping exhibited in canonical analysis of principal components (CAP analysis; tr(Q’_m_HQ_m_) = 3.00, p < 0.001; δ^2^_1_ = 0.977, p < 0.001)(Fig 9). Conducting the same tests above without juvenile *Xylophaga* did not cause any changes in the direction or magnitude of the results.

Differences in community composition were tested across site, depth, and treatment-substrate for both natural substrates and between experimental and natural rocks. Notably, substrate pairings differed significantly between experimental and natural rock sample groups when comparing only inorganic (rock) substrates (combined deployments; pseudo-F = 2.73, df = 1, p < 0.001). Pairwise tests revealed a significant difference in composition between experimental carbonate rock samples and natural basalts across sites and depths (i.e., across deployments; t = 1.58, df = 15.87, p < 0.001), which suggests an influence of treatment or substrate type beyond the effects of the organic input of wood substrates. There was also a significant difference between the natural basalt rocks found at SJS and natural basalts at 40MB (combined depths; t = 1.42, df = 11.96, p = 0.001), indicating the influence of proximity to shore on natural rock communities. Unfortunately, there were insufficient samples to conduct pairwise tests for experimental and natural rocks of the same type across deployments (i.e., ferromanganese and sedimentary rocks; df = 0), which limited our ability to parse the effect of treatment from that of environmental factors.

Depth had a major influence on the non-xylophagaid macrofaunal communities. The interaction between the treatment-substrate factor and the depth factor was significant without juvenile *Xylophaga* (combined sites; pseudo-F = 1.32, df = 6, p = 0.03). Pairwise tests indicated a significant difference in composition between deep and shallow experimental carbonate rock colonizers (combined sites; t = 1.45, df = 4, p = 0.04), between deep and shallow wood block colonizers (combined sites; t = 2.48, df = 4, p = 0.03), and between deep and shallow natural basalt rock communities (combined sites; t = 1.52, df = 10, p < 0.001). Within the substrate-treatment pairwise comparison (combined deployments), experimental sedimentary rocks differed significantly from natural basalt rocks without juvenile *Xylophaga* (t = 1.25, df = 11.08, p = 0.02). All other PERMANOVA results for this dataset remained unchanged when run without juvenile or adult *Xylophaga* included in the analysis, so while depth had a significant influence on some of the non-xylophagaid community trends, it remains a less compelling determinant for community composition than substrate type.

In line with the density results, community composition differed significantly between 2020 and 2021 on natural rocks across deployments, both with (pseudo-F = 4.01, df = 1, p < 0.001) and without (pseudo-F = 2.143, df = 1, p < 0.001) the inclusion of juvenile *Xylophaga* counts. SIMPER analysis comparing 2020 and 2021 natural rock samples revealed low in-group (10.48% for 2020, 16.18% for 2021) and between-group (3.27%) similarity. The highest contributor to between-group dissimilarity was juvenile *Xylophaga* (9.02%) found on 2021 natural rocks, followed by a serpulid morphospecies (more abundant in 2020; 3.04%) and several ophiuroid morphospecies (1.44–2.18%), most of which were more abundant in 2021. These results further suggest the outsized influence of *Xylophaga* on community structure in this study.

## Discussion

### Wood shadow effects: *Xylophaga*

Juvenile *Xylophaga* appeared only on hard substrates collected in 2021 (including natural rocks), after the 10-month deployment of the wood blocks, and not on any rocks collected in 2020 (S1 Table). This suggests that *Xylophaga* larvae were induced to settle by the presence of the experimental wood and would not have otherwise been found as juveniles on these substrates. Most wood fall (and therefore *Xylophaga*-related) studies occur away from hard-ground habitats, so observations of xylophagaid interactions with hard substrates are uncommon. However, an early study of a wood deployment at 70 m in Monterey Bay, California reported juvenile *Xylophaga washingtona* (whose descriptions matched the juveniles observed in this study) crawling on and attempting to penetrate the concrete anchor of the study’s wood deployment [25]. This indicates that juvenile *Xylophaga*, once induced to settle (e.g., by chemical cues from fecal chimneys or from the wood itself [28]), explore substrates before burrowing.

The presence of relatively high densities of juvenile *Xylophaga* on adjacent, non-wood substrates may also reflect a “mass effect” [47] in which species move from areas of high to low density. A similar observation of mass effect of juvenile *Xylophaga* was reported in Bernardino et al., 2010 [13], albeit in sediments adjacent to a large, deployed wood block. As with the juvenile *Xylophaga* found on experimental rock substrates in our experiment, Bernardino et al. (2010) found no evidence of maturation in these specimens and therefore considered them a sink population resulting from mass effects [13]. Spillover of organic specialists from the nearby wood substrates onto slate rock was also observed by Young et al. [23], but it was not reported for juvenile *Xylophaga*, nor in the high abundances observed in the present experiment (E.L. Young, *personal communication*). This difference may reflect species-level variation. Young et al. identified their specimens as *X. oregona*, which are commonly observed deeper than 1500 m [23,40]. No studies have directly compared the recruitment, boring, and growth rates of these species, but it remains likely that interspecific variation may account for the different behavior observed in this study (and others with *X. washingtona*; [13,25]) and in Young et al., 2022.

The observed mass effect of juvenile *Xylophaga* could also have been the effect of currents that picked up juveniles from experimental wood substrates and deposited them nearby, as the experiments were deployed at sloped hard-ground sites subject to potentially strong bottom currents during ebb and flood tidal phases. However, given the similar effect observed in other wood fall studies, it remains likely that this mass effect is a consistent, documented effect associated with the *Xylophaga* colonization at wood falls. Similarly, while limited contamination between substrates may have been possible during the collection of wood and rocks into the bioboxes (e.g., *Xylophaga* juveniles falling off collected wood substrates into rock containers while the biobox lid was open), the spillover effect was observed on nearly all substrates, including those that did not share a biobox with wood substrates. This, as well as the large numbers of juvenile *Xylophaga* observed on these substrates, suggests that this observation was likely not the result of contamination between samples. Ultimately, the abundances of the juvenile xylophagaid bivalves in this experiment are a unique observation with strong implications for successional stage dynamics both at and near wood falls.

Due to the absence of wood present to support juvenile *Xylophaga* on experimental rock substrates, they were likely either dead upon collection or likely to experience mortality in the near future due to starvation or predation (i.e., a “sink population,” as in Bernardino et al., 2010 [13]). The life cycles of xylophagaids remain understudied, but laboratory studies have indicated that pre-metamorphosis (i.e., pediveligers) and partially-metamorphosized *Xylophaga* may exist in a state of stasis for as long as six months, potentially as an adaptation to the boom and bust availability of wood fall habitats [27]. The pediveligers and partially-metamorphosized juveniles described in this and other early studies of *Xylophaga* match the “juveniles” observed in this experiment [25,27]. Though it is not possible to know how long the juvenile *Xylophaga* had been settled on this study’s rock substrates, the vast majority of the juveniles found were alive upon collection, with shells full of tissue and many specimens observed with small siphons out, indicating that these juveniles may have been exhibiting a similar state of stasis, and/or have been exploring nearby substrates for suitable habitat as observed by Haderlie, 1983 [25].

*Xylophaga* are well-documented as early successional stage animals and foundation species at wood falls [22,48–50], a pattern reflected in the high densities of wood specialist annelids (Fig 6) and the elevated total densities observed on our experimental wood substrates (Fig 5). However, juvenile *Xylophaga* densities were positively correlated with total densities of other taxa (mainly annelids) on experimental rock substrates (Fig 4). The juvenile *Xylophaga* on non-wood substrates (which were nonviable and presumably vulnerable to predation due to a lack of wood for nourishment and shelter) may have served as a food source for other taxa on these rock substrates. *Xylophaga* fecal pellets were found in the tubes and stomachs of multiple annelid species (*Decemunciger* sp., Dorvilleidae spp.) on experimental wood substrates, and juvenile *Xylophaga* were also found in tubes and guts (Fig 7). Though this phenomenon was only observed on the experimental wood substrates, it indicates the viability of both *Xylophaga* and its byproducts as a food source for other fauna. This finding supports hypotheses by others suggesting that ampharetid and dorvilleid polychaetes feed on xylophagaid fecal material [23]. Ampharetids are known to be selective feeders, often preferring smaller particles that are lighter, which likely includes fine organic material found in feces [51]. The presence of scraping teeth in some Ampharetidae species further supports the idea that these worms can mechanically remove and ingest soft, labile material, a feeding strategy similar to their method of collecting microbial films from surfaces [52]. Though no wood-specialist polychaetes were observed on rock substrates, several dorvilleid polychaete morphospecies were observed on both experimental carbonate rocks and wood substrates (S1 Dataset). Given the limited taxonomic certainty of these identifications (genus- to family-level identification), it is unclear whether these individuals were hard-substrate generalists or wood-associated taxa attracted by the presence of juvenile *Xylophaga* or nearby wood substrates. Future study will be necessary to determine if the wood shadow effect extends to other wood-associated taxa, but the mass effect of wood specialists and organic opportunists observed in Young et al., 2022 supports this possibility [23].

The significantly lower densities of both adult and juvenile *Xylophaga* at the shallow offshore experimental deployment (SJS 694 m) is not easily explained by oxygen concentration (which was higher at SJS than 40MB), food availability (the wood remained intact), or proximity to wood inputs. The deep offshore deployment had high densities of adult and juvenile *Xylophaga*, suggesting that this site was close enough to other, natural wood falls to be reached by larvae. The macrofaunal densities on non-wood substrates were also lower at SJS 694 m than at other deployments, with densities remaining closer to that of the natural rocks. This difference is therefore more likely to reflect environmental factors not tested in this study such as flow rates impacting larval recruitment and settlement, as in Clark et al. [53]. Any reduction in the ability of *X. washingtona* to originally colonize these wood substrates would have compounded the difference in community structure observed at this deployment vs. others, as reproduction by early *Xylophaga* colonizers likely contributed to the high juvenile *Xylophaga* densities observed at the other deployment locations. Furthermore, given the influence of *Xylophaga* on overall colonization trends at and near wood substrates, the lack of *Xylophaga* colonization may have inhibited colonization of the experimental substrates (both wood and rocks) by other fauna, as exhibited by the low densities at the shallow SJS deployment across substrate types and taxa. This is supported by the findings of Smith et al. [24], in which low initial colonization of wood blocks by these ecosystem engineers suppressed further colonization of experimental wood substrates by other fauna.

Community composition did not vary significantly by deployment (Site x Depth) in the experimental or combined datasets, both with and without juvenile *Xylophaga* included in the analyses, indicating that the unique pattern observed at the shallow SJS deployment is limited to faunal densities. The trend observed could be the result of cascading impacts from the low *Xylophaga* densities at this deployment, potentially reflecting their outsized role in colonization dynamics. Alternatively, the low densities at the shallow SJS location could be the result of environmental conditions that impacted the ability of all macrofauna (not just *Xylophaga*) to colonize substrates at this site. The trend could also be the result of different predation rates and predator densities at the different sites. Both of these possible explanations have support in Smith et al. [24], which found medium- and broad-scale changes in environmental conditions to play a significant role in the long-term colonization of wood substrates, and also saw substantial variability in mobile predator and scavenger abundance in the first several months of their colonization experiment, throughout which *Xylophaga* densities remained low. Ultimately, more site-specific research would be necessary to determine the cause and implications of this deployment location-specific trend in *Xylophaga* densities.

### Substrate type

The significantly higher macrofaunal densities observed on wood substrates is consistent with other studies of both experimental and natural wood falls in which wood provides a fleeting but nutrient-rich source of food for opportunistic animals [1,10,22]. These findings align closely with Cunha et al. [54], in which experimentally deployed wood substrates at 300 m, 700 m, and 1100 m exhibited significantly higher densities than experimentally deployed carbonate rocks. Xylophagaid bivalves are generally early colonizers of wood that make these nutrients accessible to other animals via fragmented or softened wood pieces or––more commonly––through their feces which are colonized by bacteria [49,50]. This process is consistent with the high concentration of opportunistic annelids like *Decemunciger* and dorvilleid polychaetes observed on experimental wood substrates here (including many with tubes containing xylophagaid fecal matter), as these annelids likely exploited one or both of these food sources. *Xylophaga* also contributes to localized nitrogen cycling via their nitrogen-fixing bacterial symbionts [55,56], aligning more broadly with other nitrogen-fixing microbial communities commonly found at organic falls [57,58]. It is possible that the *Xylophaga* colonizers observed on the experimental wood blocks played a similar role of enhancing nitrogen availability at the deployment sites, which in turn helped support elevated densities of nearby faunal communities (e.g., as in [13]).

Further in line with existing research, wood substrate community composition differed significantly from all other substrate types and had high within-group similarity due to dominance by wood specialists such as *X. washingtona*, *Ophryotrocha* spp., other dorvilleids, and *Decemunciger* sp., which also contributed to the low evenness observed for experimental wood substrates. Strong clustering in ordination plots was exhibited by wood substrate samples and, to a lesser extent, carbonate rock samples (Fig 9). However, the lack of significant difference in community composition between different experimental rock substrate types was not consistent with observations of substrate preferences by macrofauna for natural rocks in the SCB [9]. Substrate-specific microbial associations, predicted by Guraieb et al. [9] to be important in generating substrate preferences, may not have had time to form in the colonization experiments. Rather, the overwhelming influence of juvenile *Xylophaga* within the “shadow” of experimental wood substrates may have superseded any other substrate effects.

As hypothesized, wood substrate communities differed significantly from all natural rocks, regardless of site, depth, or year, in accordance with extant knowledge regarding distinct organic fall communities [23,50,59]. However, the significant difference between experimental carbonate rocks and multiple natural rock types including basalt, ferromanganese rock, and sedimentary rock suggests that experimental substrates may also have been exhibiting earlier successional stages than natural rocks, or that the more complex structure of carbonate rock may have provided a more desirable habitat for certain communities. On the Costa Rica margin, distinct communities developed on carbonate and wood substrates over 7.4 years at transition sites off methane seeps [15], but macrofaunal composition was similar on 7.4-year colonization carbonate deployments to that of natural carbonate rocks [14]. Due to the lack of naturally-occurring carbonate rocks at the deployment sites (and thus the inability to collect them as in-situ rocks for this study), further future study comparing experimental and in-situ carbonate rocks (e.g., at inactive methane seeps) will be necessary to determine whether the results found here were due to successional stage dynamics or due to specific properties inherent to carbonate rocks (e.g., surface texture).

### Successional stage dynamics and environmental variability

The lack of significant differences in macrofaunal densities between the two deployment depths (and therefore oxygen concentrations) indicates that substrate type may be a more powerful driver of overall macrofaunal colonization in the SCB than environmental factors, which is consistent with colonization experiments across ocean basins and with different substrates [15,23,41,60]. Oxygen-availability studies in the deep sea show that low oxygen concentrations can suppress faunal densities [24,61–63], including wood colonization by *Xylophaga* [24]. However, given the strong influence of *Xylophaga* densities on overall macrofaunal density trends, it is possible that the food and habitat created by these wood specialists may have overshadowed the impact of low oxygen concentrations at the two shallower sites.

The lack of significant variation in total macrofaunal densities on wood substrates between different depths and sites contrasts with other wood colonization experiments performed in deep-sea settings [23,48,50], in which wood substrate deployments differed significantly based on location. However, when *Xylophaga* were excluded, total non-xylophagaid invertebrate densities were found to be significantly higher inshore (at 40-mile bank). This finding is consistent with wood colonization studies which found higher macrofaunal densities closer to shore [23,48,50]. While few studies exist of non-organic hard substrate colonization in non-reducing ecosystems, trends of decreasing deep-sea macrofaunal densities with increasing distance from shore are well-documented and consistent with our findings [64–66]. The food and habitat availability created by *Xylophaga* at the inshore site––perhaps coupled with higher food availability on non-organic substrates from greater POC flux inshore––could be the dominant driver of this trend for non-xylophagaid colonists.

Though medium- and broad-scale temporal variability in environmental controls were also recently found to influence the colonization of wood and carbonate rocks on the northeast Pacific margin [24], the lack of significant difference between total macrofaunal densities observed on natural rocks in 2020 vs 2021 indicates that the higher macrofaunal densities observed on experimental substrates are likely not the result of interannual variations in environmental conditions. Though the higher densities on experimental substrates could be a result of the *Xylophaga* “shadow” as discussed above, this disparity could also indicate earlier successional stages of colonization on experimental substrates resulting from the relatively short 10-month deployment period. This is consistent with previous research in other ecosystems (e.g., sediments, methane seeps) in which deployment duration was critical in determining the density and composition of colonizers [14,67–69]. The same possible explanations may apply to the observed diversity trends, with lower diversities on experimental substrates potentially resulting from dominance or spillover by wood specialists, or from earlier successional stages, though the lack of significant difference in species richness between natural and experimental substrates may suggest the former. Replication of this experiment on longer time scales could help separate the impact of successional stage dynamics from the impact of nearby organic inputs on both density and diversity trends.

Environmental variability did play a role in shaping community composition, in line with previous colonization experiments [23,24,70]. Depth influenced community composition for experimental substrates in the present study, though the significant interaction between depth and substrate type indicates that the influence of depth may be dependent on the substrate type, as only wood substrate communities differed significantly between depths. These results remained unchanged by the removal of juvenile *Xylophaga*, and therefore are unlikely due solely to the disparity in juvenile *Xylophaga* densities between substrate types. This trend was echoed in the significantly higher species richness at deeper depths for experimental substrates, which was also driven by a highly significant difference in wood substrates between depths (higher richness at deeper depths). Though no colonizer diversity metrics differed significantly by site, within taxonomic groups, annelids and echinoderms exhibited significantly different densities between sites (Table 2). Trends in annelid densities by site largely matched those of *Xylophaga* densities, which is once again consistent with the well-documented role of *Xylophaga* in facilitating wood colonization by other taxa [22,23,49,50]. Meanwhile, higher densities of echinoderms (mostly ophiuroids) at inshore sites may have resulted from higher POC flux to the seafloor further inshore, as deep-sea ophiuroids and other echinoderms depend primarily on marine snow for food [71–73].

## Conclusion

The results of this experiment support the idea that substrate type influences the structure of deep-sea macrofaunal communities. As expected, wood fall specialists dominated wood experimental substrates. However, the extent of this influence exceeded previously published observations, with juvenile *Xylophaga* exhibiting a “spillover effect,” settling on non-wood hard substrates located near the experimental wood blocks. The positive correlation between densities of juvenile *Xylophaga* and other macrofaunal densities on rock substrates suggests that these juvenile *Xylophaga* may have influenced nearby communities through competition or resource provision. Although some taxa exhibited density or diversity patterns in line with known environmental trends (e.g., proximity to shore, depth), the spillover influence of experimental wood substrates generally overshadowed the influence of these environmental conditions, indicating that the provision of food and habitat by wood specialists may supersede the influence of oxygen concentration and POC flux on communities. Ultimately, these findings suggest that xylophagaid bivalves can have an outsized role on successional stage dynamics not just at wood falls, but within a sphere of influence or “wood shadow” surrounding wood falls. This influence merits further research to better define the extent, duration, and conditions required for this effect to take place, as it could have implications for future management of deep-sea ecosystems. These results could also imply an even stronger link between coastal forests and deep-sea biodiversity than previously recognized. As anthropogenic impacts on marine ecosystems (e.g., climate change, deoxygenation, benthic disturbance such as mining or trawling) expand and accelerate, understanding how organic inputs interact with, overshadow, or potentially insulate faunal communities from disturbance and changing environmental conditions will become increasingly important.

## Supporting information

S1 Table Xylophagaid bivalve densities on experimental and in-situ substrates, according to year, treatment, and substrate type. Includes samples from the 2021 experimental substrates (wood, carbonate rock, sedimentary rock, phosphorite rock, ferromanganese rock) and both the 2021 and 2020 natural substrates (sedimentary rock, ferromanganese rock, basalt). Samples are from both of the study’s sites (San Juan Seamount, 40-Mile Bank), each of which had two depths (~1100 m, ~ 700 m) in the Southern California Borderland.
(DOCX)

S1 Dataset Macrofaunal community data. The raw data used for the statistical analyses and data visualization of the macrofaunal communities (> 300 μm) observed on the experimental substrates (wood, carbonate rock, sedimentary rock, phosphorite rock, ferromanganese rock; collected in 2021) and natural substrates (sedimentary rock, ferromanganese rock, basalt; collected in 2020 and 2021) at both deep (~1100 m) and shallow (~700 m) deployments at San Juan Seamount and 40-Mile Bank in the Southern California Borderland.
(XLSX)
