# Supplementary material for: Wood shadows: The influence of Xylophaga on hard-substrate macrofauna in Southern California
Source: PLoS One. 2025 Dec 3;20(12):e0337217. doi: 10.1371/journal.pone.0337217 (PMC12674563; doi:10.1371/journal.pone.0337217)
Supplement: S1 Table — Includes samples from the 2021 experimental substrates (wood, carbonate rock, sedimentary rock, phosphorite rock, ferromanganese rock) and both the 2021 and 2020 natural substrates (sedimentary rock, ferromanganese rock, basalt). Samples are from both of the study’s sites (San Juan Seamount, 40-Mile Bank), each of which had two depths (~1100 m, ~ 700 m) in the Southern California Borderland. (DOCX) [file pone.0337217.s001.docx]

**S1 Table. *Xylophaga* densities on experimental and natural substrates, according to year, treatment, and substrate type.** Includes samples from the 2021 experimental substrates (wood, carbonate rock, sedimentary rock, phosphorite rock, ferromanganese rock) and both the 2021 and 2020 natural substrates (sedimentary rock, ferromanganese rock, basalt). Samples are from both of the study’s sites (San Juan Seamount, 40-Mile Bank), each of which had two depths (~1100 m, ~700 m) in the Southern California Borderland.

| Year | Treatment | Site | Depth (m) | Substrate type | Adult *X. washingtona* density (indiv/200cm^2^) | Juvenile *Xylophaga* density (indiv/200cm^2^) |
| --- | --- | --- | --- | --- | --- | --- |
| 2020 | Natural | 40-Mile Bank | 658 | Sedimentary rock | 0.00 | 0.00 |
|  |  |  | 691 | Sedimentary rock | 0.00 | 0.00 |
|  |  |  | 870 | Ferromanganese Rock | 0.00 | 0.00 |
|  |  |  |  | Ferromanganese Rock | 0.00 | 0.00 |
|  |  |  | 1036 | Sedimentary rock | 0.00 | 0.00 |
|  |  | San Juan Seamount | 691 | Basalt | 0.00 | 0.00 |
|  |  |  | 692 | Ferromanganese Rock | 0.00 | 0.00 |
|  |  |  | 954 | Ferromanganese Rock | 0.00 | 0.00 |
|  |  |  |  | Ferromanganese Rock | 0.00 | 0.00 |
|  |  |  | 1129 | Ferromanganese Rock | 0.00 | 0.00 |
| 2021 | Natural | 40-Mile Bank | 686 | Basalt | 0.00 | 0.48 |
|  |  |  | 692 | Basalt | 0.00 | 56.75 |
|  |  |  |  | Basalt | 0.00 | 5.24 |
|  |  |  |  | Sedimentary Rock | 0.00 | 1.18 |
|  |  |  | 1039 | Basalt | 0.00 | 29.09 |
|  |  |  |  | Basalt | 0.00 | 13.69 |
|  |  |  |  | Sedimentary Rock | 0.00 | 3.24 |
|  |  | San Juan Seamount | 691.3 | Basalt | 0.00 | 0.63 |
|  |  |  | 691.4 | Basalt | 0.00 | 6.22 |
|  |  |  |  | Basalt | 0.00 | 0.37 |
|  |  |  | 691.5 | Basalt | 0.00 | 0.00 |
|  |  |  | 691.6 | Basalt | 0.00 | 0.47 |
|  |  |  |  | Basalt | 0.00 | 0.35 |
|  |  |  | 1117 | Basalt | 0.00 | 0.00 |
|  |  |  |  | Basalt | 0.00 | 0.30 |
|  |  |  | 1121 | Basalt | 0.00 | 7.17 |
|  |  |  | 1121.5 | Ferromanganese Rock | 0.00 | 0.16 |
|  |  |  | 1122 | Ferromanganese Rock | 0.00 | 0.00 |
|  |  |  | 1124 | Ferromanganese Rock | 0.00 | 0.68 |
|  |  |  | 1125 | Basalt | 0.00 | 4.68 |
| 2021 | Experimental | 40-Mile Bank | 692 | Ferromanganese Rock | 0.00 | 94.88 |
|  |  |  |  | Phosphorite Rock | 0.00 | 20.21 |
|  |  |  |  | Carbonate Rock | 0.00 | 10.92 |
|  |  |  |  | Carbonate Rock | 0.00 | 154.94 |
|  |  |  |  | Wood | 1103.92 | 962.24 |
|  |  |  |  | Wood | 1278.33 | 1317.94 |
|  |  |  | 1039 | Ferromanganese Rock | 0.00 | 70.21 |
|  |  |  |  | Phosphorite Rock | 0.00 | 20.33 |
|  |  |  |  | Carbonate Rock | 0.00 | 114.26 |
|  |  |  |  | Carbonate Rock | 0.00 | 26.16 |
|  |  |  |  | Wood | 728.32 | 3635.48 |
|  |  |  |  | Wood | 615.83 | 3546.23 |
|  |  | San Juan Seamount | 694 | Sedimentary Rock | 0.00 | 1.49 |
|  |  |  |  | Carbonate Rock | 0.00 | 0.60 |
|  |  |  |  | Carbonate Rock | 0.00 | 0.21 |
|  |  |  |  | Wood | 81.26 | 6.69 |
|  |  |  |  | Wood | 34.77 | 10.60 |
|  |  |  | 1124 | Sedimentary Rock | 0.00 | 60.30 |
|  |  |  |  | Carbonate Rock | 0.00 | 0.00 |
|  |  |  |  | Carbonate Rock | 0.00 | 19.60 |
|  |  |  |  | Wood | 910.36 | 678.31 |
|  |  |  |  | Wood | 1181.46 | 3198.16 |
